# Genome-Wide Identification and Expression Analysis of JAZ Family Involved in Hormone and Abiotic Stress in Sweet Potato and Its Two Diploid Relatives

**DOI:** 10.3390/ijms22189786

**Published:** 2021-09-10

**Authors:** Zhengwei Huang, Zhen Wang, Xu Li, Shaozhen He, Qingchang Liu, Hong Zhai, Ning Zhao, Shaopei Gao, Huan Zhang

**Affiliations:** Key Laboratory of Sweet Potato Biology and Biotechnology, Ministry of Agriculture and Rural Affairs/Beijing Key Laboratory of Crop Genetic Improvement/Laboratory of Crop Heterosis & Utilization and Joint Laboratory for International Cooperation in Crop Molecular Breeding, Ministry of Education, College of Agronomy & Biotechnology, China Agricultural University, Beijing 100193, China; Huangzwxyy@163.com (Z.H.); wangzhen555@cau.edu.cn (Z.W.); nongdalixu@126.com (X.L.); sunnynba@cau.edu.cn (S.H.); liuqc@cau.edu.cn (Q.L.); zhaihong@cau.edu.cn (H.Z.); zhaoning2012@cau.edu.cn (N.Z.); spgao@cau.edu.cn (S.G.)

**Keywords:** sweet potato, *Ipomoea trifida*, *Ipomoea triloba*, *JAZ*, tissue-specific expression, hormone treatment, abiotic stress

## Abstract

Jasmonate ZIM-domain (JAZ) proteins are key repressors of a jasmonic acid signaling pathway. They play essential roles in the regulation of plant growth and development, as well as environmental stress responses. However, this gene family has not been explored in sweet potato. In this study, we identified 14, 15, and 14 *JAZs* in cultivated hexaploid sweet potato (*Ipomoea batatas*, 2n = 6x = 90), and its two diploid relatives *Ipomoea trifida* (2n = 2x = 30) and *Ipomoea triloba* (2n = 2x = 30), respectively. These *JAZs* were divided into five subgroups according to their phylogenetic relationships with *Arabidopsis*. The protein physiological properties, chromosome localization, phylogenetic relationship, gene structure, promoter *cis*-elements, protein interaction network, and expression pattern of these 43 *JAZs* were systematically investigated. The results suggested that there was a differentiation between homologous *JAZs*, and each *JAZ* gene played different vital roles in growth and development, hormone crosstalk, and abiotic stress response between sweet potato and its two diploid relatives. Our work provided comprehensive comparison and understanding of the *JAZ* genes in sweet potato and its two diploid relatives, supplied a theoretical foundation for their functional study, and further facilitated the molecular breeding of sweet potato.

## 1. Introduction

Jasmonic acid (JA) plays a central role in plant growth, developmental processes, and plant response to biotic and abiotic stresses [1,2,3,4]. The crosstalks among JA and other plant hormones regulate the balance between plant growth and defense [5,6,7]. The core JA signaling module consists of the JA receptor CORONATINE INSENSITIVE 1 (COI1), which interacts with multiple proteins to form the SCF^COI1^ E3 ubiquitin ligase complex, a subset of jasmonate–ZIM domain (JAZ) repressor proteins, and the various transcription factors such as MYC2 involved in regulating the expression of JA-responsive genes. In the absence of JA-Ile, the bioactive form of JA, *JAZ* proteins act as repressors of the transcription factors; however, the presence of JA-Ile promotes the interaction between COI1 and *JAZ* proteins, and the *JAZ* proteins are subsequently ubiquitinated by SCF^COI1^ and degraded through the 26S proteasome pathway. Thus, the repression of the transcription factors is relieved, allowing them to activate the expression of JA-responsive genes [8,9,10,11,12]. Therefore, the *JAZ* repressor is critical in the signaling cascades triggered by jasmonates.

The *JAZ* family is a branch of the TIFY family that has a conserved TIF[F/Y]XG motif known as the TIFY (ZIM) domain [13]. Besides, *JAZs* contain a Jas (CCT2) domain with consensus sequences SLX_2_FX_2_KRX_2_RX_5_PY near its C terminal [14]. The different domains provide specific interactions for proteins. The TIFY domain could recruit TOPLESS (TPL) protein by interacting with the NOVEL INTERACTOR OF *JAZ* (NINJA) proteins, which contain ERF-associated amphiphilic repression (EAR) motif, and then contribute to transcriptional repression of jasmonate responses [15,16,17]. The Jas domain could interact with COI1 and transcription factors that were involved in JA signaling pathway [18]. The *Arabidopsis*, maize, rice, *Brassica napus*, *Camellia sinensis*, and soybean genomes encode 13, 26, 15, 52, 13, and 25 *JAZs*, respectively [13,19,20,21,22,23]. The *JAZ* proteins from different plant species could be divided naturally into different numbers of sub-groups based on their evolutionary relationship [20,21,22,23]. Recent studies have shown that individual JAZ participated in abiotic stress and hormone treatment responses. In *Arabidopsis*, *JAZ*1/4 could interact with ICE1/4 to repress freezing stress [24]. WRKY57 interacted with *JAZ*4/8 and IAA29 and was involved in JA-induced leaf senescence signaling pathways mediated by auxin and JA [25]. *JAZ1* protein interacted with DELLA proteins which are repressors of the gibberellin (GA) pathway, to mediate the crosstalk of JA and GA [26]. Several *JAZ* repressors could interact with ABSCISIC ACID INSENSITIVE 3 (ABI3) to integrate JA and abscisic acid (ABA) signaling pathways during seeding germination [27]. The effector HopX1 from *Pseudomonas syringae* interacted with and promoted the degradation of *JAZ* proteins by regulating JA and salicylic acid (SA) signaling pathways [28]. In rice, more efficient activation of *OsJAZ8*, which was driven by the *ZOS3–11* promoter, was accompanied by improved salt tolerance of the transgenic seedlings [29]. OsJAZ1 interacted with OsbHLH148 to enhance drought tolerance [30]. In soybean, GmWRKY40 interacted with *JAZ* proteins and response to *Phytophthora sojae* by modulating hydrogen peroxidase accumulation [31]. In apple, JAZ1–TRB1–MYB9 module dynamically modulated JA-mediated accumulation of anthocyanin and proanthocyanidin. However, only *IbJAZ10* was characterized in sweet potato. Heterologous expression of *IbJAZ10* decreased fusarium wilt resistance in tobacco [32]. Therefore, more research needs to be conducted on *JAZs* of sweet potato.

Sweet potato (*Ipomoea batatas* (L.) Lam., 2n = B_1_B_1_B_2_B_2_B_2_B_2_ = 6x = 90), belonging to the family Convolvulaceae, is an important food source for both humans and domesticated animals, as well as a new source of bioenergy in the form of bioethanol for fuel production [33]. It is widely planted in more than 100 countries or regions worldwide [34]. As sweet potato is mainly planted on marginal land, it is routinely exposed to suboptimal growth conditions, such as drought [35,36], salinity [37,38], low temperature [39,40], and oxidation [41,42], underscoring the importance of improving abiotic tolerance to maintain productivity. The sweet potato genome is large and complex due to polyploidy and a high degree of heterozygosity. In recent years, genome assemblies of a hexaploid sweet potato Taizhong 6 [43], and two diploid species closely related to the hexaploid sweet potato, *Ipomoea trifida* NCNSP0306 (2n = 2x = 30) and *Ipomoea triloba* NCNSP0323 (2n = 2x = 30) [44], were released, making it possible to identify and analyze important gene families at the whole genome level in sweet potato.

In this study, a total of 43 *JAZs* (i.e., 14 in *I. batatas*, 15 in *I. trifida*, and 14 in *I. triloba*) were identified from the cultivated hexaploid sweet potato and its two diploid relatives. They were classified into five subgroups. We systematically investigated the properties of the protein, chromosome localization, phylogenetic relationship, conserved motifs, gene structure, *cis*-elements of the promoter, and protein interaction network of *JAZs* in sweet potato. Moreover, the tissue specificity and expression pattern analysis for hormone response and abiotic stress of *JAZs* were analyzed by qRT-PCR and RNA-seq. The evolution, different functions on development, hormone crosstalk, and abiotic stress response were discovered between sweet potato and its two diploid relatives.

## 2. Results

### 2.1. Identification and Characteristic of JAZs in Sweet Potato and Its Two Diploid Relatives

In order to identify all *JAZs* in sweet potato and its two diploid relatives, three typical strategies (i.e., Blastp search, hmmersearch, SMART and CD-search databases) were employed. A total of 43 *JAZs* were identified in *I. batatas* (14), named after “*Ib*”; *I. trifida* (15), named after “*Itf*”; and *I. triloba* (14), named after “*Itb*”. The physicochemical properties of *JAZs* were analyzed using the sequences from *I. batatas* (Table 1). The CDS length of *IbJAZs* ranges from 637 bp (*IbJAZ8.2*) to 2118 bp (*IbJAZ4*), and the genomic lengths vary from 918 bp to 4050 bp. The amino acid lengths of IbJAZs range from 138 aa (*IbJAZ8.2*) to 384 aa (*IbJAZ4*), molecular weight (MW) varies from 15.585 kDa to 40.743 kDa, and isoelectric point (pI) distributes from 7.82 (*IbJAZ1.4*) to 9.83 (*IbJAZ8.2*). The *IbJAZs* are alkaline (pI > 7) and contain more than one phosphorylation site. Up to 86% of the *IbJAZs* contain more Ser phosphorylation sites than Thr or Tyr phosphorylation sites. Most of the *IbJAZs* are unstable, with an instability index of more than 43. The GRAVY scores of *IbJAZs* are less than 0, which indicates that they are hydrophilic proteins. Subcellular localization prediction assay showed that most of the *IbJAZs* were located in the cytoplasm, but also in microbody (*IbJAZ3.2*), mitochondrial (*IbJAZ8.1* and *IbJAZ10.2*), and nucleus (*IbJAZ9*). The three-dimensional structural models of *IbJAZs* with homology higher than 20% were predicted and showed in Figures S1 and S2. The result showed that all IbJAZs have α-helices, and four *IbJAZs* (*IbJAZ3.2*, *IbJAZ6.1*, *IbJAZ10.1*, and *IbJAZ10.2*) have β-sheets.

**Figure 1:**
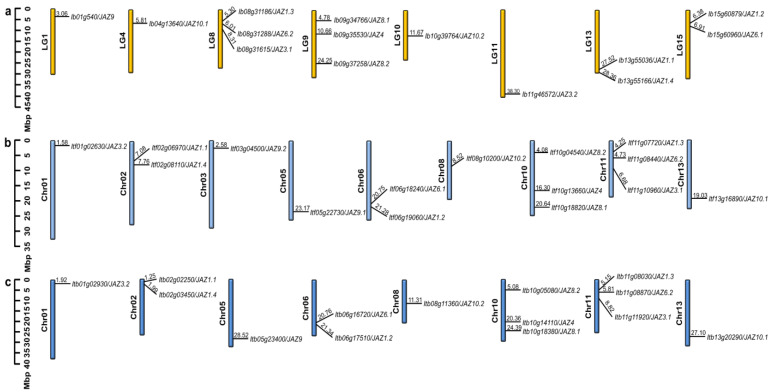
Chromosomal localization and distribution of *JAZ* genes in *I. batatas* (**a**), *I. trifida* (**b**), and *I. triloba* (**c**). The bars represent chromosomes, the chromosome numbers are displayed on the left side, and the gene names are displayed on the right side. The relative chromosomal localization of each *JAZ* gene is marked on the black line of the right side and indicated by the unit Mbp. Details chromosomal location information is listed in Table S1.

All the *JAZs* were separately mapped on 15 chromosomes of *I. batatas*, *I. trifida*, and *I. triloba*, and dispersed on 8, 9, and 8 chromosomes (Figure 1). In *I. batatas*, three *IbJAZs* were detected on LG8 and LG9, two on LG13 and LG15, one on LG1, LG4, LG10, and LG11, whereas no gene was detected on LG2, LG3, LG5, LG6, LG7, LG12, and LG14 (Figure 1a). In *I. trifida* and *I. triloba*, except *ItfJAZ9.2* was located on Chr03 only in *I. trifida,* the distribution of other *JAZs* was similar. Three *JAZs* were detected on Chr10 and Chr11; two on Chr02 and Chr06; and one on Chr01, Chr05, Chr08, and Chr13 (Figure 1b,c). The results indicated that the distribution of *JAZs* is different and disproportionate on chromosomes in sweet potato and its two diploid relatives.

### 2.2. Phylogenetic Relationship of JAZs in Sweet Potato and Its Two Diploid Relatives

**Figure 2:**
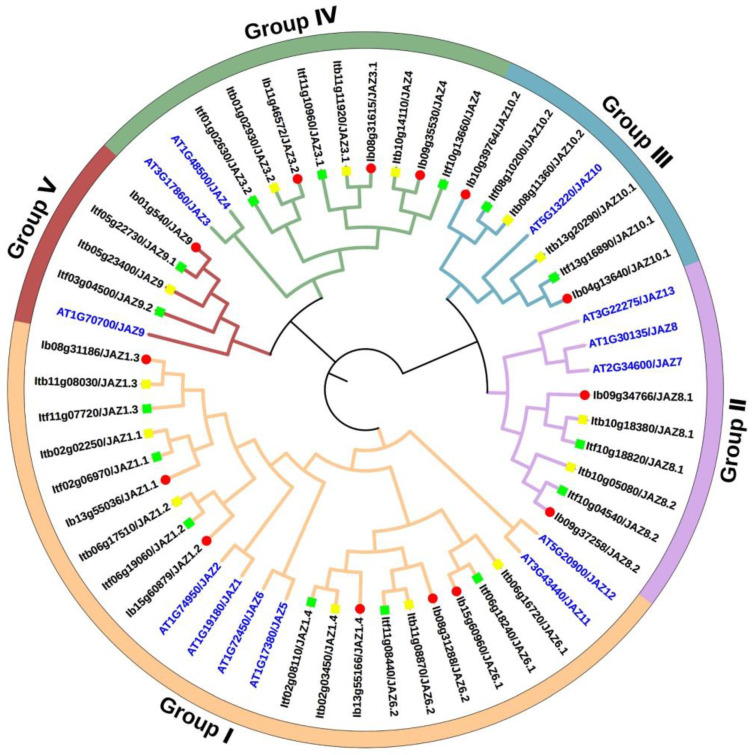
Phylogenetic analysis of the JAZ family in *I. batatas*, *I. trifida*, *I. triloba*, and *Arabidopsis*. A total of 56 JAZs were divided into five subgroups (Group I to V) according to the evolutionary distance. The red circles represent the 14 *IbJAZs* in *I. batatas*. The green squares represent the 15 *ItfJAZs* in *I. trifida*. The yellow squares represent the 14 *ItbJAZs* in *I. triloba*. The blue letters represent the 13 *AtJAZs* in *Arabidopsis*.

To study the evolutionary relationship of *JAZs* in *I. batatas*, *I. trifada*, *I. triloba*, and *Arabidopsis*, we constructed a phylogenetic tree for 56 JAZs of these four species (i.e., 14 in *I. batatas*, 15 in *I. trifida*, 14 in *I. triloba*, and 13 in *Arabidopsis*) (Figure 2). All *JAZs* were unevenly distributed, and they were divided into five subgroups (Group I to V) according to the evolutionary distance. The specific distribution of *JAZs* was as follows (total: *I. batatas*, *I. trifida*, *I. triloba*, *Arabidopsis*): Group I (24: 6, 6, 6, 6), Group II (9: 2, 2, 2, 3), Group III (7: 2, 2, 2, 1), Group IV (11: 3, 3, 3, 2), and Group V (5: 1, 2, 1, 1) (Figure 2; Table S1). We named *IbJAZs*, *ItfJAZs*, and *ItbJAZs* based on their homology with homologs in *Arabidopsis*, and only AtJAZ1/3/4/6/8/9/10 from *Arabidopsis* have homologous protein in *I. batatas*, *I. trifada*, and *I. triloba*. These results indicated that the number and type of *JAZs* distributed in each subgroup in sweet potato were similar with its two diploid relatives but different with *Arabidopsis*.

Furthermore, a total of 93 *JAZ* proteins from seven plant species (i.e., 13 in *Arabidopsis*, 9 in *Beta vulgaris*, 13 in *C. sinensis*, 15 in rice, 14 in *I. batatas*, 15 in *I. trifida*, and 14 in *I. triloba*) were used for the phylogenetic analysis. They were divided naturally into 10 sub-groups (G1 to G10) (Figure S3). The *JAZs* in *I. batatas*, *I. trifida* and *I. triloba* were specifically distributed in G1 (3, 3, 3), G2 (3, 3, 3), G5 (3, 3, 3), G8 (1, 2, 1), G9 (2, 2, 2), and G10 (2, 2, 2) (Figure S3). However, the G7 group had the maximal number of BvJAZs, the G6 group had the maximal number of CsJAZs, and the G3 and G4 groups had the maximal number of OsJAZs (Figure S3). These results may indicate that sweet potato and its two diploid relatives had a distant evolutionary relationship with *B. vulgaris*, *C. sinensis*, and rice.

### 2.3. Conserved Motif and Exon-Intron Structure Analysis of JAZs in Sweet Potato and Its Two Diploid Relatives

**Figure 3:**
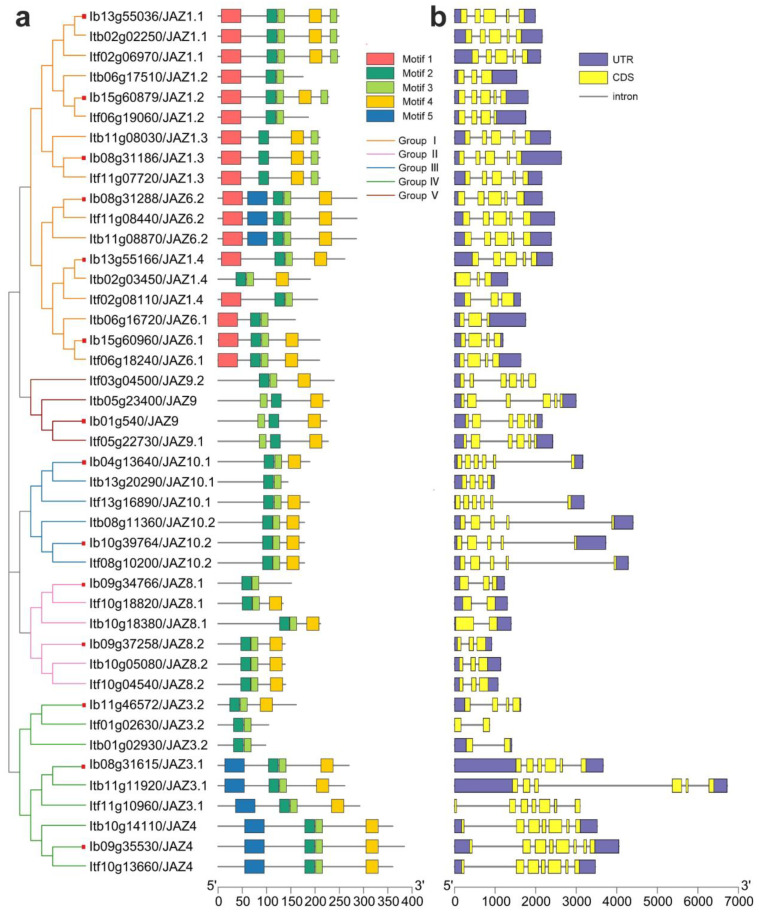
Conserved motifs and exon–intron structure analysis of JAZ family in *I. batatas*, *I. trifida, and I. triloba.* (**a**) The phylogenetic tree showed that JAZs were distributed to five subgroups in the left, and the five conserved motifs were shown in different colors. The red squares represent the IbJAZs. (**b**) Exon–intron structures of *JAZs*. The purple boxes, yellow boxes, and black lines represent the UTRs, exons, and introns, respectively.

The 43 *JAZs* from *I. batatas*, *I. trifida,* and *I. triloba* were subjected to conserved motif analysis using the MEME website, and the five most conserved motifs were identified (Figure 3a and Figure S4a). We found that *JAZs* in the same subgroup have similar conserved motifs, whereas there were differences in the types of motifs between each subgroup. For example, motifs 1 contained NT domains, and they specifically exist in Group I; motifs 2 and motifs 3 contained TIFY domains, motifs 4 contained Jas domains, and they were found in almost all *JAZs*; motifs 5 were uncertainly domains and mainly located to the N-terminal in Group IV (Figure 3a). The seqLogos of the five most conserved motifs generated using all *JAZs* sequences revealed that these motifs were conserved in sweet potato and its two diploid relatives (Figure S4a). Next, we focused on the multiple sequence alignment of *JAZ* proteins in *I. batatas* and *Arabidopsis* (Figure S4b). The TIFY domains had a conserved T(I/V)FYXG sequence, and the Jas domains had a conserved SLX_2_FLXKRKXRX_5_PY sequence. They were relatively conserved in *I. batatas* and *Arabidopsis*. In addition, we found that even though the *JAZs* are highly homologous in *I. batatas*, *I. trifida* and *I. triloba*, they may differ in the number and species of conserved domains, such as *IbJAZ1.2* (contained motifs 1, 2, 3, 4, and 5) and *ItfJAZ1.2* (contained motifs 1, 2, and 3) in Group I, *IbJAZ8.1* (contained motifs 2 and 3) and *ItfJAZ8.1* (contained motifs 2, 3, and 4) in Group II, *IbJAZ10.1* (contained motifs 2, 3, and 4) and *ItbJAZ10.1* (contained motifs 2 and 3) in Group III, and *IbJAZ3.2* (contained motifs 2, 3, and 4) and *ItfJAZ3.2* (contained motifs 2 and 3) in Group IV (Figure 3a).

To better understand the structural diversity among the *JAZs*, the exon–intron structures were analyzed (Figure 3b). The number of exons in the *JAZs* ranged from 2 to 8. In detail, *JAZs* of Group I contained 3 to 5 exons, *JAZs* of Group II contained 2 or 3 exons, *JAZs* of Group III contained 4 to 6 exons, *JAZs* of Group IV contained 2 to 8 exons, and *JAZs* of Group V contained six exons (Figure 3b). We also found that the exon–intron structures of some homologous *JAZs* might be different in *I. batatas* compared with that in *I. trifida* and *I. triloba*, such as *IbJAZ1.2* (contained five exons) and *ItfJAZ1.2* (contained four exons); in Group I, IbJAZ8.1 (contained three exons) and *ItfJAZ8.1* (contained two exons); in Group II, *IbJAZ10.1* (contained six exons) and *ItbJAZ10.1* (contained four exons); in Group III, and *IbJAZ3.2* (contained four exons) and *ItfJAZ3.2* (contained two exons); and in Group IV(Figure 3b). The results show that the *JAZ* gene family may have undergone a lineage-specific differentiation event in the sweet potato genome.

### 2.4. Cis-Element Analysis in the Promoter of IbJAZs in Sweet Potato

**Figure 4:**
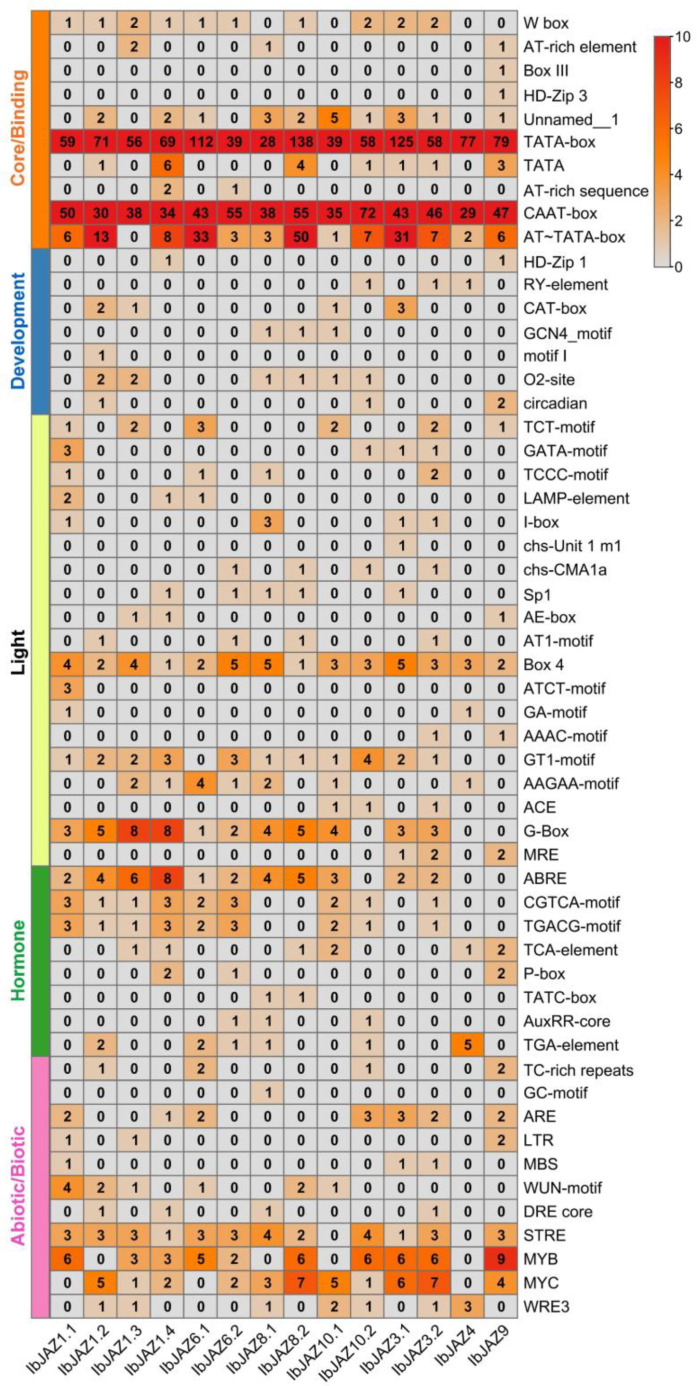
*Cis*-elements analysis of *IbJAZ*s in *I. batatas*. The *cis*-elements were divided into five broad categories. The degree of red colors represents the number of *cis*-elements upstream of the *IbJAZs*.

*Cis*-elements present in the upstream of *JAZs* play important roles in the gene functions involved in plant development and stress responses. Therefore, we extracted 2000 bp upstream sequences of *IbJAZs* in *I. batatas* and performed the *cis*-element analysis. According to the prediction function, the elements were grouped into five broad categories: core/binding, development, light, hormone, abiotic/biotic elements (Figure 4). All 14 *JAZs* possessed a large number of core promoter elements and binding sites, such as TATA-box, CAAT-box, and AT-TATA-box. Light-responsive elements, such as BOX4, GT1-motif, G-box, were founded in most of *IbJAZs*.

Moreover, some development-related elements were found in *IbJAZs* (Figure 4). For example, motif I, which was related to the root growth, was found only in *IbJAZ1.2*; the CAT-box, which was associated with meristem formation and cell division, was found in *IbJAZ1.2*, *IbJAZ1.3*, *IbJAZ3.1*, and *IbJAZ10.1*. Besides, hormonal-responsive elements were abundant in *IbJAZs* (Figure 4), including ABA-responsive element ABRE, MeJA-responsive elements CGTCA-motif and TGACG-motif, SA-responsive element TCA, GA-responsive elements P-box and TATC-box, and IAA-responsive elements AuxRR-core and TGA-element. In addition, some abiotic-responsive elements (Figure 4), such as drought-responsive element DRE core, MYB and MYC, and low temperature-responsive element LTR, were found in most *JAZs*. These results suggested that *IbJAZs* were involved in the regulation of plant growth and development and abiotic stress adaption in sweet potato.

### 2.5. Protein Interaction Network of IbJAZs in Sweet Potato

**Figure 5:**
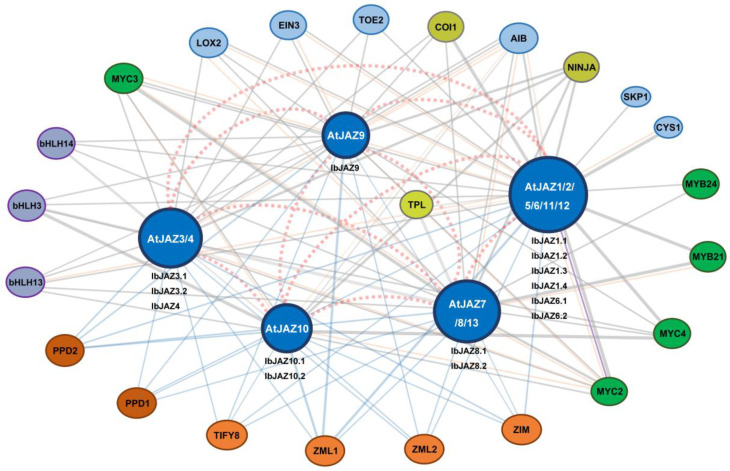
Functional interaction networks of *IbJAZ* in *I. batatas* according to orthologues in Arabidopsis. Network nodes represent proteins, and lines represent protein–protein associations. The red dotted lines represent the strong interactions of *JAZs* with each other. The gray lines represent physical interactions. The blue lines represent shared protein domains. The orange lines represent co-expression. The purple lines represent co-localization. The line thickness represents the strength of the interaction.

To explore the potential regulatory network of *IbJAZs*, we constructed an *IbJAZs* interaction network based on *Arabidopsis* orthologous proteins (Figure 5). Protein interaction prediction indicated that *IbJAZs* could interact with each other. They also interacted with TIFY family proteins (i.e., PPD1, PPD2, ZIM, ZML1, ZML2, and TIFY8). In addition, *IbJAZs* could interact with numerous transcription factor families proteins, such as bHLH (i.e., bHLH3, bHLH13, and bHLH14), MYC (i.e., MYC2, MYC3, and MYC4), MYB (MYB21 and MYB24), and AP2 (i.e., TOE2), and some hormone synthesis and signaling related proteins, such as jasmonate biosynthesis-related protein lipoxygenase 2 (LOX2) and ethylene signaling related protein ethylene insensitive 3 (EIN3). These results indicated that IbJAZs might participate in the hormone regulation network through interacting with related transcription factors and functional proteins.

### 2.6. Expression Analysis of JAZs in Sweet Potato and Its Two Diploid Relatives

#### 2.6.1. Expression Analysis in Various Tissues

**Figure 6:**
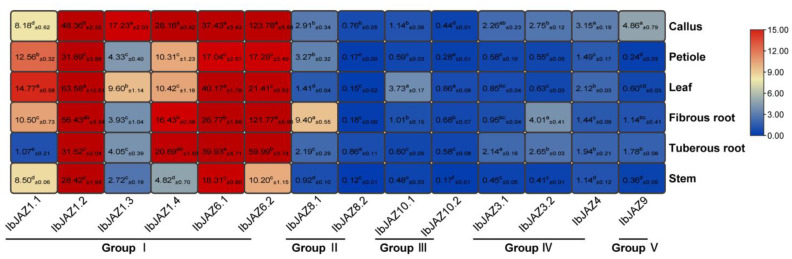
Gene expression patterns of *IbJAZs* in different tissues (callus, petiole, leaf, fibrous root, tuberous root, and stem) of *I. batatas.* The values were determined by RT-qPCR from three biological replicates consisting of pools of three plants, and the results were analyzed using the comparative C_T_ method. The expression of *IbJAZ10.1* in fibrous root was considered as “1”. Fold change ± SD was shown in the boxes. Different lowercase letters indicate a significant difference of each *IbJAZ* at *p* < 0.05 based on Student’s *t*-test.

To investigate the potential biological functions of *IbJAZs* in growth and development, the expression levels in six representative tissues (i.e., callus, petiole, leaf, fibrous root, tuberous root, and stem) of *I. batatas* were analyzed using real-time quantitative PCR (qRT-PCR) (Figure 6). Interestingly, different subgroups showed diversified expression patterns in six tissues. *IbJAZs* in Group I showed higher expression levels in all tissues than in other subgroups. Among all the *IbJAZs*, three *IbJAZs* (i.e., *IbJAZ1.2*, *IbJAZ6.1*, and *IbJAZ6.2*) were highly expressed, whereas two *IbJAZs* (i.e., *IbJAZ8.2* and *IbJAZ10.2*) were low-expressed in all tissues. Nevertheless, some *IbJAZ* showed tissue-specific expression, e.g., *IbJAZ8.1* was highly expressed in fibrous root, *IbJAZ10.1* was highly expressed in leaf, *IbJAZ3.2* was highly expressed in roots, *IbJAZ9* was highly expressed in callus, but *IbJAZ1.1* was lowly expressed in the tuberous root. These results indicated that *IbJAZs* might play different roles in the tissue development of sweet potato.

In addition, we used RNA-seq data of seven tissues (i.e., callus, flower, flower bud, leaf, root 1, root 2, and stem) to study the expression patterns of *ItfJAZs* and *ItbJAZs* in *I. trifida* and *I. triloba* [44] (Figure S5). *ItfJAZs* and *ItbJAZs* in Group I showed higher expression levels than other subgroups, which was consistent with that in *I. batatats*. In *I. trifida*, six *ItfJAZs* (i.e., *ItfJAZ1.1*, *ItfJAZ1.3*, *ItfJAZ6.1*, *ItfJAZ6.2*, *ItfJAZ8.1*, and *ItfJAZ10.1*) were highly expressed, whereas *ItfJAZ3.2* was lowly expressed in all tissues. Besides, *ItfJAZ1.2* and *ItfJAZ1.4* were lowly expressed in flower and flower bud, three *ItfJAZs* (i.e., *ItfJAZ3.1*, *ItfJAZ9.1*, and *ItfJAZ9.2*) were lowly expressed in root, *ItfJAZ4* was lowly expressed in root and stem, *ItfJAZ8.2* was lowly expressed in callus, and *ItfJAZ10.2* was highly expressed in leaf, root, and stem (Figure S5a). In *I. triloba*, eight *ItbJAZs* (i.e., *ItbJAZ1.1*, *ItbJAZ1.3*, *ItbJAZ1.4*, *ItbJAZ4*, *ItbJAZ6.2*, *ItbJAZ8.1*, *ItbJAZ8.2*, and *ItbJAZ9*) were highly expressed, but three *ItbJAZs* (i.e., *ItbJAZ3.1*, *ItbJAZ3.2*, and *ItbJAZ10.1*) were lowly expressed in all tissues. Besides, *ItbJAZ1.2* was lowly expressed in flower, *ItbJAZ6.1* was lowly expressed in leaf, and *ItbJAZ10.2* was highly expressed in root (Figure S5b). These results suggested that each *JAZ* gene had a different tissue expression pattern and played different regulatory roles in the growth and development of sweet potato and its two diploid relatives.

#### 2.6.2. Expression Analysis of Hormone Response

**Figure 7:**
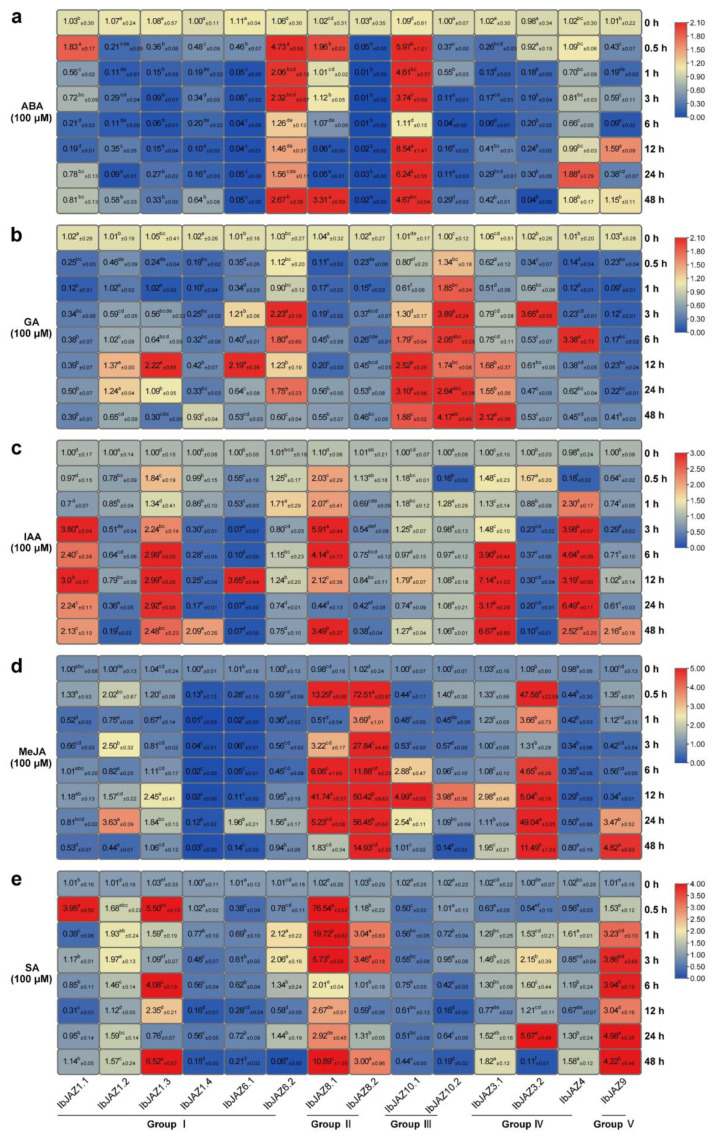
Gene expression patterns of *IbJAZs* in response to different phytohormone, i.e. (**a**) ABA, (**b**) GA, (**c**) IAA, (**d**) MeJA, and (**e**) SA of *I. batatas.* The values were determined by RT-qPCR from three biological replicates consisting of pools of three plants, and the results were analyzed using the comparative C_T_ method. The expression of 0 h in each treatment was considered as “1”. The fold change ± SD was shown in the boxes. Different lowercase letters indicate a significant difference of each *IbJAZ* at *p* < 0.05 based on Student’s *t*-test.

The *JAZ* family plays a crucial role in the hormone signal transduction and crosstalk of plants. Thus, it is necessary to investigate the expression of *JAZs* under various hormonal treatments. We performed qRT-PCR to evaluate the expression level of *IbJAZs* in response to the hormone, including ABA, GA, IAA, MeJA, and SA. Under ABA treatment, most of *IbJAZs* were repressed, but two *IbJAZs* (i.e., *IbJAZ6.2* and *IbJAZ10.1*) were significantly induced (Figure 7a). Under GA treatment, more than half of *IbJAZs* were significantly induced, specifically *IbJAZ6.2*, *IbJAZ10.1,* and *IbJAZ10.2* were up-regulated, but five *IbJAZs* (i.e., *IbJAZ1.1*, *IbJAZ1.4*, *IbJAZ8.1*, *IbJAZ8.2*, and *IbJAZ9*) were repressed at most of the time points (Figure 7b). Under IAA treatment, six *IbJAZs* (i.e., *IbJAZ1.1*, *IbJAZ1.3*, *IbJAZ3.1*, *IbJAZ4*, *IbJAZ6.1*, and *IbJAZ8.1*) were significantly induced, but four *IbJAZs* (i.e., *IbJAZ1.2*, *IbJAZ1.4*, *IbJAZ3.2*, and *IbJAZ8.2*) were repressed (Figure 7c). Under MeJA treatment, most of *IbJAZs* were up-regulated, specifically *IbJAZ3.2, IbJAZ8.1*, and *IbJAZ8.2* were significantly induced by 49-, 41-, and 72-fold, but two *IbJAZs* (i.e., *IbJAZ1.4* and *IbJAZ4*) were repressed (Figure 7d). Under SA treatment, six *IbJAZs* (i.e., *IbJAZ1.1*, *IbJAZ1.3*, *IbJAZ3.2*, *IbJAZ8.1*, *IbJAZ8.2*, and *IbJAZ9*) were significantly up-regulated, specifically *IbJAZ8.1* was significantly induced by 76-fold at 0.5 h, but four *IbJAZs* (i.e., *IbJAZ1.4*, *IbJAZ6.1*, *IbJAZ10.1*, and *IbJAZ10.2*) were repressed (Figure 7e). Among these *IbJAZs*, *IbJAZ1.4* was down-regulated in almost all the five hormone treatments, whereas other *IbJAZs* were induced by two or more hormones. These results indicated that *IbJAZs* were differentially expressed in response to various hormone induction and participated in the crosstalk between various hormones.

In addition, we analyzed the expression patterns of *ItfJAZs* and *ItbJAZs* using the RNA-seq data of *I. trifida* and *I. triloba* under ABA, GA, and IAA treatments [44]. In *I. trifida*, compared with hormone control, *ItfJAZ1.2* was induced by ABA and IAA treatments, whereas *ItfJAZ1.4* was repressed under all ABA, GA, and IAA treatments (Figure S6a). In *I. triloba*, compared with hormone control, *ItbJAZ6.1* was induced by all ABA, GA, and IAA treatments (Figure S6b). Most other *JAZs* respond to at least one hormone treatment in *I. trifida* and *I. triloba*. Furthermore, compared to the expression patterns of *IbJAZs* in sweet potato (Figure 7), the homologous *JAZs* in *I. trifida* and *I. triloba* responded differently to ABA, GA, and IAA treatments, which indicated that *JAZs* were involved in different hormonal pathways between sweet potato and its two diploid relatives.

#### 2.6.3. Expression Analysis under Abiotic Stresses

**Figure 8:**
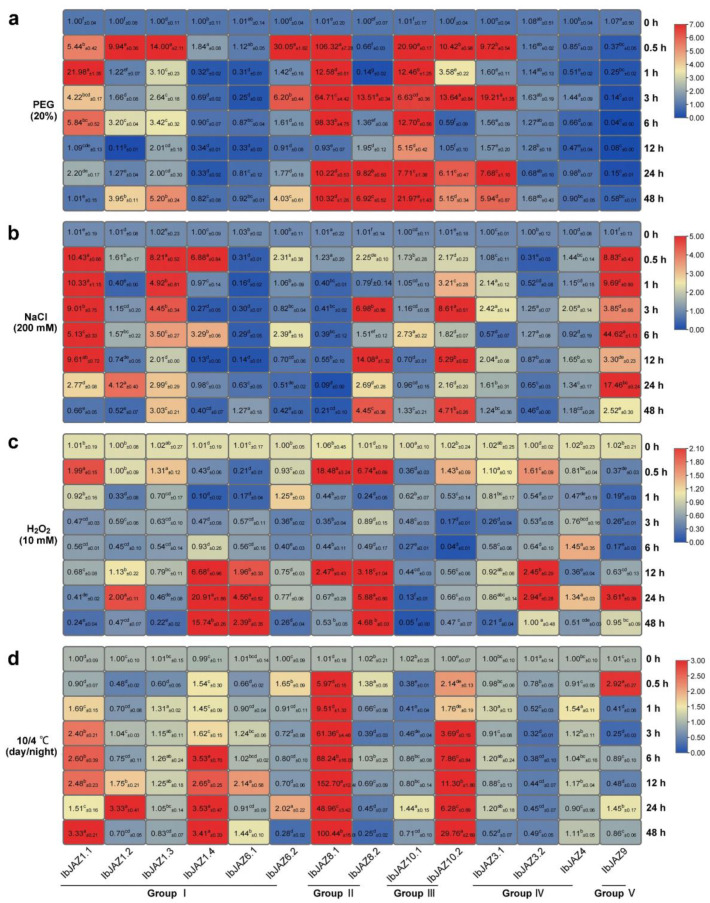
Gene expression patterns of *IbJAZs* in response to abiotic stresses, i.e. (**a**) PEG, (**b**) NaCl, (**c**) H_2_O_2_, and (**d**) 10/4 °C treatments of *I. batatas.* The values were determined by RT-qPCR from three biological replicates consisting of pools of three plants, and the results were analyzed using the comparative C_T_ method. The expression of 0 h in each treatment was considered as “1”. The fold change ± SD was shown in the boxes. Different lowercase letters indicate a significant difference of each *IbJAZ* at *p* < 0.05 based on Student’s *t*-test.

To explore the possible roles of *IbJAZs* in abiotic stress response, we analyzed the expression level of *IbJAZs* under polyethylene glycol (PEG), NaCl, H_2_O_2_, and 10/4 ℃ (day/night) treatments by qRT-PCR (Figure 8). Under PEG treatment, most of *IbJAZs* were significantly up-regulated with *IbJAZ8.1* particularly induced by 106-fold at 0.5 h, but four *IbJAZs* (i.e., *IbJAZ1.4*, *IbJAZ4*, *IbJAZ6.1*, and *IbJAZ9*) were repressed (Figure 8a). Under NaCl treatment, some *IbJAZs* (e.g., *IbJAZ1.1*, *IbJAZ1.2*, *IbJAZ1.3*, *IbJAZ1.4*, *IbJAZ8.2*, *IbJAZ9*, and *IbJAZ10.2*) were significantly up-regulated with *IbJAZ9* particularly induced by 44-fold at 6 h, but *IbJAZ8.1* was repressed (Figure 8b). Under H_2_O_2_ treatment, some *IbJAZs* (e.g., *IbJAZ1.4*, *IbJAZ6.1*, *IbJAZ8.1*, and *IbJAZ8.2*) were significantly induced, whereas *IbJAZ10.1* was repressed (Figure 8c). Under 10/4 ℃ (day/night) treatment, some *IbJAZs* (i.e., *IbJAZ1.1*, *IbJAZ1.4*, *IbJAZ8.1*, and *IbJAZ10.2*) were significantly up-regulated, specifically *IbJAZ8.1* was induced by 152-fold at 12 h, whereas *IbJAZ3.2* was significantly repressed (Figure 8d). Among these *IbJAZs*, several *IbJAZs*, such as *IbJAZ1.2* and *IbJAZ* 10.2, were induced by all the four abiotic stress treatments in sweet potato.

**Figure 9:**
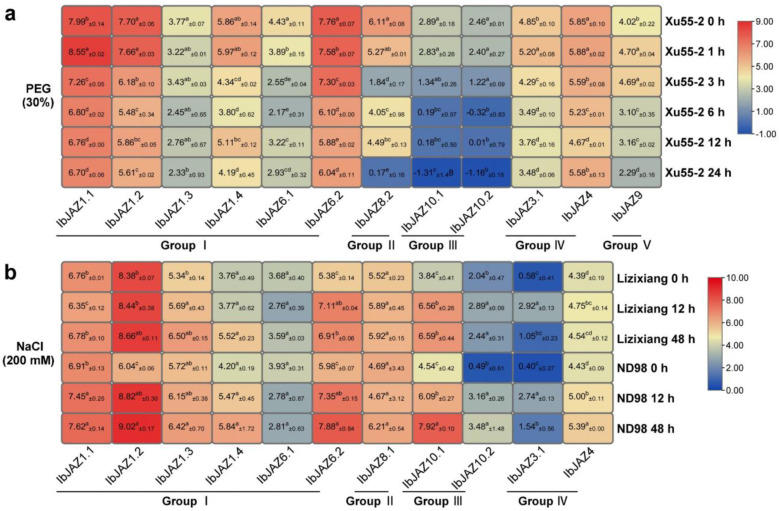
Gene expression patterns of *IbJAZs* under PEG and NaCl treatments as determined by RNA-seq. (**a**) Expression analysis of *IbJAZs* under PEG treatment in a drought-tolerant variety Xu55-2. (**b**) Expression analysis of *IbJAZs* under NaCl treatment in a salt-sensitive variety Lizixiang and a salt-tolerant line ND98. Log2 (FPKM ± SD) was shown in the boxes. Different lowercase letters indicate a significant difference of each *IbJAZ* at *p* < 0.05 based on Student’s *t*-test.

Moreover, we analyzed the expression patterns of *IbJAZs* using the RNA-seq data of a drought-tolerant variety Xu55-2 under PEG treatment and the RNA-seq data of a salt-sensitive variety Lizixiang and a salt-tolerant line ND98 under NaCl treatment [45,46]. Consistent with qRT-PCR results (Figure 8), the expression of *IbJAZ6.1* was also repressed in Xu55-2 under PEG treatment (Figure 9a), and the expression of some *IbJAZs* (e.g., *IbJAZ1.3*, *IbJAZ1.4*, and *IbJAZ10.2*) were induced in ND98 under NaCl treatment (Figure 9b). Excluding *IbJAZ1.2* and *IbJAZ10.2*, expression patterns of other *IbJAZs* were similar in both Lizixiang and ND98 under NaCl treatment (Figure 9b). Taken together, these results indicated that *IbJAZs* were differentially expressed in response to various abiotic stresses in sweet potato.

In addition, we also analyzed the expression patterns of *ItfJAZs* and *ItbJAZs* using the RNA-seq data of *I. trifida* and *I. triloba* under mannitol, NaCl, and 10/4 °C (day/night) treatments [44]. In *I. trifida*, compared with control, *ItfJAZ1.4* and *ItfJAZ8.2* were induced by mannitol and 10/4 °C (day/night) treatments, and *ItfJAZ9.2* was induced by NaCl treatment (Figure S7a). In *I. triloba*, compared with control, *ItbJAZ1.2*, *ItbJAZ3.1*, and *ItbJAZ8.2* were induced, whereas *ItbJAZ3.2* and *ItbJAZ9* were repressed by mannitol, NaCl, and 10/4 °C (day/night) treatments (Figure S7b). Moreover, compared to the expression patterns of *IbJAZs* in sweet potato, the homologous *JAZs* in *I. trifida* and *I. triloba* responded differently to mannitol, NaCl, and 10/4 °C (day/night) treatments, which indicated that *JAZs* were involved in different abiotic stress responses between sweet potato and its two diploid relatives.

## 3. Discussion

*JAZ* repressors were universally reported to participate in plant growth and development, playing hub roles in hormonal crosstalk and responding to environmental stresses and biotic challenges [47]. However, the functional roles of *JAZ* family genes are still poorly understood in sweet potato. As the genetic background of cultivated sweet potato is complex, previous studies on the sweet potato gene families mainly focused on its most probable progenitor diploids *I. trifida* [48,49,50,51]. In fact, the plant morphology of cultivated hexaploid sweet potato differs greatly from that of its diploid relatives, especially since its diploid relatives cannot form tuberous roots [44]. In this study, we systematically identified *JAZ* family genes and analyzed and compared their characteristics based on the draft genome sequence of cultivated hexaploid sweet potato and its two diploid relatives. Genome-wild study of *JAZs* plays an important guiding role in the further study of their function and molecular breeding of sweet potato.

### 3.1. Evolution of the JAZ Gene Family in Sweet Potato and Its Two Diploid Relatives

In this study, a total of 43 *JAZs* were identified from sweet potato and its two diploid relatives. The number of *JAZs* identified in *I. batatas* (14) is the same as *I. triloba* (14) (Figure 1 and Figure 2; Table S1) but is one less than that in *I. trifida* (15). These numbers were also similar to that of other higher plants, such as *Arabidopsis* (13), rice (15), and wheat (14) [13,22,52]. This result indicated that the *JAZ* gene members in sweet potato are relatively conserved during the evolutionary process. Genomic alignment reveals the differentiation and evolution of chromosomes [53]. The chromosome localization and distribution of *JAZs* in each chromosome were different between *I. batatas*, *I. trifida*, and *I. triloba* (Figure 1). Based on the phylogenetic relationship, *JAZs* are divided into five subgroups (Group I to V), and only AtJAZ1/3/4/6/8/9/10 from *Arabidopsis* found homologous proteins in *I. batatas*, *I. trifada*, *I. triloba*. Except for Group V, the number of *JAZs* distributed in each subgroup was the same in Group I to IV among *I. batatas*, *I. trifida* and *I. triloba*. However, the number and type of *JAZs* distributed in each subgroup of sweet potato and its two diploid relatives were different from those of *Arabidopsis* and other plants (Figure 2 and Figure S3). These results showed that the *JAZ* gene family might have undergone a lineage-specific differentiation event in the terrestrial plant genome.

In plants, a small number of introns evolve more quickly to respond to environmental changes, whereas the introns usually act as buffer zones or mutation-resistant fragments that reduce adverse mutations and insertions. Moreover, introns also play essential roles in mRNA export, transcriptional coupling, alternative splicing, gene expression regulation, and other biological processes [54,55]. We found that the exon–intron structures of some homologous *JAZs* might be different in *I. batatas* compared with that in *I. trifida* and *I. triloba*, and the number of exons and introns of *I. batatas* was generally higher than that of *I. trifida* and *I. triloba*. For example, *IbJAZ1.2* (contained five exons) and its homologous gene *ItfJAZ1.2* (contained four exons) in Group I, *IbJAZ8.1* (contained three exons) and its homologous gene *ItfJAZ8.1* (contained two exons) in Group II, *IbJAZ10.1* (contained six exons) and its homologous gene *ItbJAZ10.1* (contained four exons) in Group III, and *IbJAZ3.2* (contained four exons) and its homologous gene *ItfJAZ3.2* (contained two exons) in Group IV (Figure 3). Therefore, homologous *JAZs* with more exon and introns in hexaploid sweet potato are evolutionally more complex than its two diploid relatives and thus participate in more precise growth and development and response to environmental stress.

In addition, the five most conserved motifs were identified from all 43 JAZs, and these motifs were highly conserved in sweet potato and its two diploid relatives (Figure 3a and Figure S4a). The same subgroup of *JAZs* usually has similar conserved motifs, whereas the types of motifs were generally different between each subgroup. The two core TIFY and Jas domains, which play irreplaceable roles in the JA signaling pathway, were present in almost all 43 *JAZs* from *I. batatas*, *I. trifida*, and *I. triloba.* While motif 1 specifically existed in Group I, and motif 5 was mainly located to the N-terminal in Group IV (Figure 3a). Therefore, *JAZs* with different motifs differentiated into a variety of biological functions during plant life activities in sweet potato.

### 3.2. Different Functions of JAZs on Growth and Development between Sweet Potato and Its Two Diploid Relatives

*JAZ* proteins play essential roles in plant growth and development. In *Arabidopsis*, AtCOI1-AtJAZ9 interaction mediated root growth inhibition [56,57]. AtJAZ7 and AtJAZ10 regulated leaf senescence through dark induction [58]. In rice, overexpression of *OsJAZ13* activated hypersensitive cell death response and resulted in root attenuation [59]. In tomato, overexpression of *SlJAZ2* led to leaf initiation, lateral bud emergence, and flowering transition [60]. Here, the *cis*-element prediction analysis showed that *IbJAZ1.2* contained a root growth-related element motif I, and *IbJAZ1.2*, *IbJAZ1.3*, *IbJAZ3.1*, and *IbJAZ10.1* contained meristem formation and cell division-related element CAT-box (Figure 4). To further explore the functions on growth and development of *JAZs*, we analyzed their expression level in the representative tissues of sweet potato and its two diploid relatives. The results indicated that *JAZ* family was differentially and constitutively expressed. The *JAZs* in Group I showed relatively high levels in all tissues compared to other subgroups of *I. batatas*, *I. trifida* and *I. triloba* (Figure 6 and Figure S5). *IbJAZ1.2,* which contained a motif I and a CAT-box, was highly expressed in fibrous and tuberous root, but *ItfJAZ1.2* and *ItbJAZ1.2* were expressed most highly in the stem and root, indicating that *IbJAZ1.2* might play regulatory roles in tuberous root development of sweet potato. Moreover, *IbJAZ10.1,* which contained a CAT-box, was only highly expressed in leaf, but *ItfJAZ10.1* and *ItbJAZ10.1* were most highly expressed in the stem, indicating that *IbJAZ10.1* might be involved in leaf development in sweet potato. The plant morphology of cultivated hexaploid sweet potato differs greatly from that of its diploid relatives, especially since its diploid relatives cannot form tuberous roots [44]. Different tissue expression patterns of homologous *JAZ* genes may contribute to tissue diversification in sweet potato and its two diploid relatives.

### 3.3. Different Functions of JAZs on Hormone Crosstalk between Sweet Potato and Its Two Diploid Relatives

Multiple interacting hormone pathways play a major role in many biological processes, including plant growth, development, and defense against a wide range of both biotic and abiotic stresses [6]. In *Arabidopsis*, *JAZ9* inhibited the interaction of RGA (a DELLA protein) with the transcription factor PIF3 and formed a COI1-JAZ-DELLA-PIF signaling module to regulate plant defense and growth by interfering with JA and GA signaling cascade [61]. AtERF109 activity was inhibited by direct interaction with *JAZ* proteins to prevent hypersensitivity to wounding, which led to JA and IAA signaling crosstalk [62]. In wheat, TaJAZ1 interacted with TaABI5 to connect the signaling between JA and ABA and modulate seed germination [63]. In grape, *VqJAZ4* enhanced disease defense responses through JA and SA signaling pathways [64]. In this study, protein interaction prediction showed that *IbJAZs* could interact with each other and also interacted with some hormone synthesis and signaling related proteins to participate in multiple hormone pathways, including TIFY family proteins PPD1, PPD2, ZIM, ZML1, ZML2, and TIFY8, jasmonate biosynthesis-related protein LOX2, and ethylene signaling related protein EIN3 (Figure 5). In addition, most of *IbJAZs* were induced by two or more hormones (Figure 7). *IbJAZ1.3* and *IbJAZ3.2*, which contained JA-responsive elements CGTCA-motif and TGACG-motif, were induced by GA, MeJA, and SA treatment, whereas *ItbJAZ3.2* was induced by IAA treatment. *IbJAZ4* and *IbJAZ6.1*, which contained IAA-responsive element TGA, were significantly induced under GA and IAA treatments, whereas *ItbJAZ6.1* was induced under ABA, GA, and IAA treatments. *IbJAZ6.2*, which contained ABA-responsive element ABRE, GA-responsive element P-box, and JA-responsive elements CGTCA-motif and TGACG-motif, was highly induced by ABA, GA, and MeJA, whereas *ItfJAZ6.2* and *ItbJAZ6.2* showed no significant change. *IbJAZ8.2* and *IbJAZ9*, which contained an SA-responsive element TCA-element, were highly expressed under MeJA and SA treatments, whereas *ItbJAZ8.2* was highly expressed under ABA treatment. *IbJAZ10.1*, which contained ABA-responsive element ABRE and JA-responsive elements CGTCA-motif and TGACG-motif, was significantly induced by ABA and MeJA treatment, whereas *ItbJAZ10.1* was induced by GA treatment. These results indicated that *JAZs* participated in multiple hormones crosstalk, and homologous *JAZ* genes were involved in different hormonal pathways in sweet potato and its two diploid relatives.

### 3.4. Different Functions of JAZs on Abiotic Stress Response between Sweet Potato and Its Two Diploid Relatives

*JAZ* proteins directly interact with or repress many TFs to modulate abiotic stress tolerance efficiently. In cotton, GbWRKY1 could function as a negative regulator of ABA signaling via *JAZ1* and ABI1, with effects on salt and drought tolerance [65]. In soybean, GmWRKY40 interacted with eight *JAZ* proteins and regulated reactive oxygen species accumulation [31]. In banana, MaJAZ1 attenuated the transcriptional activation of MaAOC2 and modulated cold tolerance [66]. In apple, MdbHLH1 interacted with MdJAZ1/4 and MdMYC2, which could bind to the *MdCBF1* promoter, to enhance cold tolerance [67]. Here, protein interaction prediction showed that *IbJAZs* could interact with numerous transcription factors, such as bHLH3/13/14, MYC2/3/4, MYB21/24, and TOE2 [68], to participate in growth and development and biotic and abiotic stresses in sweet potato.

In addition, *IbJAZs* significantly responded to various abiotic stresses in sweet potato (Figure 8 and Figure 9). *IbJAZ3.1*, containing drought-responsive element MYC, was highly induced by PEG treatment at most of the time points; *IbJAZ1.1*, *IbJAZ1.3*, *IbJAZ1.4*, *IbJAZ9*, and *IbJAZ10.2* were highly induced by NaCl treatment; *IbJAZ1.4*, *IbJAZ6.1*, *IbJAZ8.1*, and *IbJAZ8.2* were highly induced by H_2_O_2_ treatment; and *IbJAZ1.1* containing a cold-responsive element LTR, *IbJAZ1.4*, *IbJAZ8.1,* and *IbJAZ10.2* were highly induced by 10/4 ℃ (day/night) treatment, suggesting that they might be involved in drought, salt, oxidation, and cold tolerance of sweet potato, respectively (Figure 8).

The results from the qRT-PCR and RNA-seq data analysis of sweet potato and its two diploid relatives under abiotic stress indicated that homologous *JAZs* in *I. trifida* and *I. triloba* responded differently compared to that of sweet potato (Figure S7). This means that *JAZs* were involved in different abiotic stress responses between sweet potato and its two diploid relatives. The diploid *I. trifida* and *I. triloba* could be used to discover functional genes, particularly genes conferring resistance or tolerance to biotic and abiotic stresses, which had been possibly lost in the cultivated sweet potato during its domestication [69]. In this study, 15 *ItfJAZs* were identified in *I. trifida*, which was one more (*ItfJAZ9.2*) than that in sweet potato. *ItfJAZ9.2* was induced by NaCl treatment. Furthermore, *ItfJAZ1.4* and *ItfJAZ8.2* were induced by mannitol and 10/4 °C (day/night) treatments (Figure S7a). *ItbJAZ1.2*, *ItbJAZ3.1*, and *ItbJAZ8.2* were induced by all mannitol, NaCl, and 10/4 °C (day/night) treatments (Figure S7b). These *JAZs* may serve as candidate genes for use in improving abiotic stress tolerance in sweet potato.

## 4. Materials and Methods

### 4.1. Identification of JAZs 

The whole genome sequences of *I. batatas*, *I. trifida,* and *I. triloba* were downloaded from *Ipomoea* Genome Hub (https://ipomoea-genome.org/, accessed on 15 March 2021) and Sweetpotato Genomics Resource (http://sweetpotato.plantbiology.msu.edu/, accessed on 15 March 2021). To accurately identify all *JAZs* family members, three different screening methods were combined. Firstly, the BLAST algorithm was used to identify predicted *JAZs* using all *AtJAZs* from the *Arabidopsis* genome database (https://www.arabidopsis.org/, accessed on 15 March 2021) as queries (BLASTP, E value ≤ 1 × 10^−5^). Next, the HMMER 3.0 software (Harvard University, Cambridge, MA, USA) was used to identify potential *JAZs* through the Hidden Markov Model profiles (hmmsearch, E value ≤ 1 × 10^−5^) of the TIFY domain (PF06200) and Jas domain (PF09425), which were extracted from the Pfam databases (http://pfam.xfam.org/, accessed on 15 March 2021). Finally, all putative JAZs were ensured using SMART (http://smart.embl-heidelberg.de/, accessed on 16 March 2021) and CD-search (https://www.ncbi.nlm.nih.gov/Structure/cdd/wrpsb.cgi, accessed on 16 March 2021).

### 4.2. Chromosomal Distribution of JAZs

The *IbJAZs*, *ItfJAZs*, and *ItlJAZs* were separately mapped to the *I. batatas*, *I. trifida,* and *I. triloba* chromosome based on the chromosomal location provided in the *Ipomoea* Genome Hub (https://ipomoea-genome.org/, accessed on 15 March 2021) and Sweetpotato Genomics Resource (http://sweetpotato.plantbiology.msu.edu/, accessed on 15 March 2021). The visualization was generated by the TBtools software (South China Agricultural University, Guangzhou, China).

### 4.3. Protein Properties Prediction of JAZs

The MW, theoretical pI, unstable index, hydrophilic of *JAZs* were calculated by ExPASy (https://www.expasy.org/, accessed on 20 March 2021). The phosphorylation sites of *JAZs* were predicted using Kinase Phos (http://kinasephos.mbc.nctu.edu.tw/, accessed on 20 March 2021). The subcellular localization of *JAZs* was predicted by PSORT (https://wolfpsort.hgc.jp/, accessed on 20 March 2021). The 3D structural model of *JAZs* was built using SWISS-MODEL (https://swissmodel.expasy.org/, accessed on 20 March 2021) [70].

### 4.4. Phylogenetic Analysis of JAZs

The phylogenetic analysis of *JAZs* from *Arabidopsis*, *Beta vulgaris*, *C. sinensis*, rice, *I. batatas*, *I. trifida*, and *I. triloba* was performed using ClustalW in MEGA 7.0 (Temple University, Philadelphia, USA) [71] with default parameters, maximum likelihood method, and Poisson correction model were used. The bootstrap was performed with 1000 replicates. Then, the phylogenetic tree was constructed by iTOL (http://itol.embl.de/, accessed on 1 September 2021). The protein sequences were downloaded from the National Center for Biotechnology Information (https://www.ncbi.nlm.nih.gov/, accessed on 31 August 2021).

### 4.5. Domain Identification and Conserved Motifs Analysis of JAZs

The conserved motifs of JAZs were analyzed by MEME (https://meme-suite.org/meme/, accessed on 18 March 2021), the maximum number of motifs parameter was set to 5. The multiple sequence alignment of the TIFY and Jas domains was built using the DNAMAN software (lynnonBiosoft, San Ramon, CA, USA).

### 4.6. Exon–Intron Structures and Promoter Analysis of JAZs

The exon–intron structures of *JAZs* were obtained by GSDS 2.0 (Peking University, Beijing, China) (http://gsds.gao-lab.org/, accessed on 19 March 2021) and were visualized by the TBtools software (South China Agricultural University, Guangzhou, China). The *cis*-elements in the approximately 2000 bp promoter region of *JAZs* were predicted by PlantCARE (http://bioinformatics.psb.ugent.be/webtools/plantcare/html/, accessed on 21 March 2021) [72].

### 4.7. Protein Interaction Network of JAZs

The protein interaction network of JAZs was predicted by GeneMAINA (http://genemania.org/, accessed on 31 August 2021) and String (https://www.string-db.org/, medium confidence 0.400, accessed on 31 August 2021) based on *Arabidopsis* orthologous proteins. The network map was built by Cytoscape software 3.2 (Institute for Systems Biology, Seattle, WA, USA) [73].

### 4.8. qRT-PCR Analysis of JAZs

The salt-tolerant sweet potato (*I. batatas*) line ‘ND98′ was used for qRT-PCR analysis in this study [46]. In vitro-grown ND98 plants were cultured on Murashige and Skoog (MS) medium at 27 ± 1 °C under a photoperiod consisting of 13 h of cool-white fluorescent light at 54 μmol m^–2^ s^–1^ and 11 h of darkness. Sweet potato plants were cultivated in a field at the campus of China Agricultural University, Beijing, China.

For expression analysis in various tissues, total RNA was extracted from the fibrous root, tuberous root, stems, leaves, petioles, and bud tissues of 3-month-old field-grown ND98 plants using the TRIzol method (Invitrogen, Carlsbad, CA, USA). For expression analysis of hormone and abiotic treatment, the leaves were sampled at 0, 0.5, 1, 3, 6, 12, 24, and 48 h after treated with 200 mM NaCl, 20% PEG6000, 10 mM H_2_O_2_, 10/4 ℃ (day/night), 100 μM ABA, 100 μM GA, 100 μM MeJA, 100 μM SA, and 100 μM IAA, respectively. Three independent biological replicates were taken, each with three plants. qRT-PCR was conducted using the SYBR detection protocol (TaKaRa, Kyoto, Japan) on a 7500 Real-Time PCR system (Applied Biosystems, Foster City, CA, USA). The reaction mixture was composed of first-strand cDNA, primer mix, and SYBR Green M Mix (TaKaRa; code RR420A) to a final volume of 20 μL. A sweet potato actin gene (GenBank AY905538, 20, 5, 21) was used as an internal control. The relative gene expression levels were quantified with the comparative C_T_ method [74]. The specific primers of qRT-PCR analysis were listed in Table S2. The heat maps of gene expression profiles were constructed using TBtools software. 

### 4.9. Transcriptome Analysis

The RNA-seq data of *ItfJAZs* and *ItlJAZs* in *I. trifida* and *I. triloba* were downloaded from the Sweetpotato Genomics Resource (http://sweetpotato.plantbiology.msu.edu/, accessed on 12 April 2021). The RNA-seq data of *IbJAZs* in *I. batatas* were obtained from related research in our laboratory [45,46]. The expression levels of JAZs were calculated as fragments per kilobase of exon per million fragments mapped (FPKM). The heat maps were constructed by TBtools software.

## 5. Conclusions

In this study, we identified and characterized 43 *JAZs* in cultivated hexaploid sweet potato (*I. batatas*, 2n = 6x = 90, 14), and its two diploid relatives *I. trifida* (2n = 2x = 30, 15) and *I. triloba* (2n = 2x = 30, 14) based on genome and transcriptome data. The protein physiological properties, chromosome localization, phylogenetic relationship, gene structure, promoter *cis*-elements, and protein interaction network of these 43 *JAZs* were systematically investigated. Moreover, the tissue specificity and expression pattern analysis for hormone response and abiotic stress of *JAZs* were analyzed by qRT-PCR and RNA-seq. Our results indicated that there was a differentiation in roles played by homologous *JAZs*, and each *JAZ* gene played different vital roles in growth and development, hormone crosstalk, and abiotic stress response between sweet potato and its two diploid relatives. This work provided valuable insights into the structure and function of *JAZ* genes in sweet potato and its two diploid relatives.

## Figures and Tables

**Table 1:** Characterization of *IbJAZs* in sweet potato.

Gene ID	Gene Name	CDS (bp)	Protein (aa)	Genomic (bp)	MW (kDa)	pI	Phosphorylation Site			Instability	Gravy	Subcellular
							Ser	Thr	Tyr			
Ib13g55036	*IbJAZ1.1*	1175	249	1990	26.730	9.56	1	1	0	43.47	−0.549	cytoplasm
Ib15g60879	*IbJAZ1.2*	1334	228	1812	25.112	9.73	4	0	0	52.26	−0.553	cytoplasm
Ib08g31186	*IbJAZ1.3*	1728	210	2633	23.110	8.81	4	1	1	47.07	−0.469	cytoplasm
Ib13g55166	*IbJAZ1.4*	1069	261	2411	27.945	7.82	5	0	0	49.55	−0.634	cytoplasm
Ib08g31615	*IbJAZ3.1*	1492	270	3663	281.33	9.72	3	0	0	58.99	−0.047	cytoplasm
Ib11g46572	*IbJAZ3.2*	759	161	1629	177.19	9.10	4	0	0	48.82	−0.074	microbody
Ib09g35530	*IbJAZ4*	2118	384	4050	40.743	9.52	6	3	0	52.80	−0.069	cytoplasm
Ib15g60960	*IbJAZ6.1*	847	210	1193	22.687	8.76	4	1	0	49.85	−0.587	cytoplasm
Ib08g31288	*IbJAZ6.2*	1400	268	2164	30.268	8.85	7	0	0	54.40	−0.605	cytoplasm
Ib09g34766	*IbJAZ8.1*	778	151	1229	17.007	8.62	5	0	0	80.04	−0.340	mitochondrial
Ib09g37258	*IbJAZ8.2*	637	138	918	15.585	9.83	2	0	0	78.45	−0.430	cytoplasm
Ib01g540	*IbJAZ9*	1074	224	2162	246.59	9.10	2	2	1	64.44	−0.448	nucleus
Ib04g13640	*IbJAZ10.1*	837	189	3163	20.718	8.86	4	0	0	66.19	−0.372	cytoplasm
Ib10g39764	*IbJAZ10.2*	1317	178	3728	19.356	9.39	6	0	0	69.18	−0.299	mitochondrial

CDS, coding sequence; MW, molecular weight; pI, isoelectric point; Ser, serine; Thr, threonine; Tyr, tyrosine.

## Data Availability

The data presented in this study are available on request from thecorresponding author.

## Supplementary Materials

The following are available online at https://www.mdpi.com/article/10.3390/ijms22189786/s1.
