# Supplementary material for: Genome-Wide Identification and Expression Analysis of JAZ Family Involved in Hormone and Abiotic Stress in Sweet Potato and Its Two Diploid Relatives
Source: Int J Mol Sci. 2021 Sep 10;22(18):9786. doi: 10.3390/ijms22189786 (PMC8468994; doi:10.3390/ijms22189786)
Supplement: Supplementary file 1 [file ijms-22-09786-s001.zip › ijms-1353783-SI.pdf]

Table S1. Identification of *JAZ* family genes in *I. batatas*, *I. trifida*, and *I. triloba*.

Arabidopsis	Homologous gene in *Ipomoea batatas/ Ipomoea trifida/ Ipomoea triloba*	Gene ID	Gene name	Chromosome localization
Group Ⅰ：AT1G19180/JAZ1 AT1G74950/JAZ2 AT1G17380/JAZ5 AT1G72450/JAZ6 AT3G43440/JAZ11 AT5G20900/JAZ12	*Ipomoea batatas*	Ib13g55036	*IbJAZ1.1*	LG13:27521003-27522993
		Ib15g60879	*IbJAZ1.2*	LG15:6376142-6377954
		Ib08g31186	*IbJAZ1.3*	LG8:5297392-5300025
		Ib13g55166	*IbJAZ1.4*	LG13:28358587-28360998
		Ib15g60960	*IbJAZ6.1*	LG15:6910297-6911490
		Ib08g31288	*IbJAZ6.2*	LG8:6009732-6011896
	Ipomoea trifida	Itf02g06970	*ItfJAZ1.1*	Chr02:7084927-7087049
		Itf06g19060	*ItfJAZ1.2*	Chr06:21283462-21285218
		Itf11g07720	*ItfJAZ1.3*	Chr11:4250574-4252729
		Itf02g08110	*ItfJAZ1.4*	Chr02:7760432-7762058
		Itf06g18240	*ItfJAZ6.1*	Chr06:20753935-20755570
		Itf11g08440	*ItfJAZ6.2*	Chr11:4732099-4734567
	Ipomoea triloba	Itb02g02250	*ItbJAZ1.1*	Chr02:1252019-1254182
		Itb06g17510	*ItbJAZ1.2*	Chr06:21344098-21345631
		Itb11g08030	*ItbJAZ1.3*	Chr11:5150719-5153083
		Itb02g03450	*ItbJAZ1.4*	Chr02:1993907-1995215
		Itb06g16720	*ItbJAZ6.1*	Chr06:20763611-20765365
		Itb11g08870	*ItbJAZ6.2*	Chr11:5805225-5807610
Group Ⅱ：AT2G34600/JAZ7 AT1G30135/JAZ8 AT3G22275/JAZ13	*Ipomoea batatas*	Ib09g34766	*IbJAZ8.1*	LG9:4781049-4782278
		Ib09g37258	*IbJAZ8.2*	LG9:24244160-24245078
	Ipomoea trifida	Itf10g18820	*ItfJAZ8.1*	Chr10:20641391-20642695
		Itf10g04540	*ItfJAZ8.2*	Chr10:4076300-4077372
	Ipomoea triloba	Itb10g18380	*ItbJAZ8.1*	Chr10:24385667-24387062
		Itb10g05080	*ItbJAZ8.2*	Chr10:5077723-5078860
Group Ⅲ：AT5G13220/JAZ10	*Ipomoea batatas*	Ib04g13640	*IbJAZ10.1*	LG4:5809948-5813111
		Ib10g39764	*IbJAZ10.2*	LG10:11663600-11667328
	Ipomoea trifida	Itf13g16890	*ItfJAZ10.1*	Chr13:19029505-19032698
		Itf08g10200	*ItfJAZ10.2*	Chr08:8518837-8523120
	Ipomoea triloba	Itb13g20290	*ItbJAZ10.1*	Chr13:27102716-27103693
		Itb08g11360	*ItbJAZ10.2*	Chr08:11307725-11312125
Group Ⅳ：AT3G17860/JAZ3 AT1G48500/JAZ4	*Ipomoea batatas*	Ib08g31615	*IbJAZ3.1*	LG8:8305614-8309277
		Ib11g46572	*IbJAZ3.2*	LG11:38298462-38300091
		Ib09g35530	*IbJAZ4*	LG9:10655036-10659086
	Ipomoea trifida	Itf11g10960	*ItfJAZ3.1*	Chr11:6682115-6685209
		Itf01g02630	*ItfJAZ3.2*	Chr01:1577796-1578656
		Itf10g13660	*ItfJAZ4*	Chr10:16300329-16303799
	Ipomoea triloba	Itb11g11920	*ItbJAZ3.1*	Chr11:8821271-8827991
		Itb01g02930	*ItbJAZ3.2*	Chr01:1921685-1920278
		Itb10g14110	*ItbJAZ4*	Chr10:20363190-20366707
Group Ⅴ：AT1G70700/JAZ9	*Ipomoea batatas*	Ib01g540	*IbJAZ9*	LG1:3059015-3061177
	Ipomoea trifida	Itf05g22730	*ItfJAZ9.1*	Chr05:23170756-23173175
		Itf03g04500	*ItfJAZ9.2*	Chr03:2577404-2579401
	Ipomoea triloba	Itb05g23400	*ItbJAZ9*	Chr05:28517160-2851416

Table S2. Primers used in this study.

Gene	Forward Primer	Reverse Primer
IbJAZ1.1	CAATACTCCCACCTTGGTGTCTC	GGGGACAACCTTAGGAACAGC
IbJAZ1.2	ATGATGAGCAGTCCCAAGGC	GACAGGAAAATGGGCACCC
IbJAZ1.3	CAACACCCAAACCCCCAAC	GCAAGTCAGAATAGGCTCTCTGG
IbJAZ1.4	AAGCCATCAGGGCACTTCG	AACACTAGATCCTGCAGGCGA
IbJAZ3.1	CTCAAGGAGCGGTTTTACCAA	CGATCCTGCAGCAGACTGTG
IbJAZ3.2	GAGTTGTTTGTCGGCTGCG	TCTGTTGGAAATTTTCCGGC
IbJAZ4	CTCCAGTTCAGGCGCCTTTA	GGGCTGGGGTGTCTTCAAA
IbJAZ6.1	GTCCATAACCACCACTCTGCC	GCAGCTCATTTCCAGAATTGG
IbJAZ6.2	CAGAGTGCTCGTGGCCTTTT	AGTGGGAACCACAGCAGCAC
IbJAZ8.1	GAACGAAGAAGACCACAAGAACAG	ATACGCTTTGCAGAGATCTCTTCA
IbJAZ8.2	AAGAAACTGCGGTTTGGAGC	TCCAACTTGGGGTGTTGTTGA
IbJAZ9	GGAATTTCACCCCAAATTCCT	GAACGGCTGTAAGTCCCCAA
IbJAZ10.1	CTTCCGCGATATTCAGCATG	CTCCTTCGAATTCGCAGAGG
IbJAZ10.2	TCGAACTCGATTTCTTCCGTC	AAATGGCACGGAAGCTTCTC
actin	AGCAGCATGAAGATTAAGGTTGTAGCAC	TGGAAAATTAGAAGCACTTCCTGTGAAC

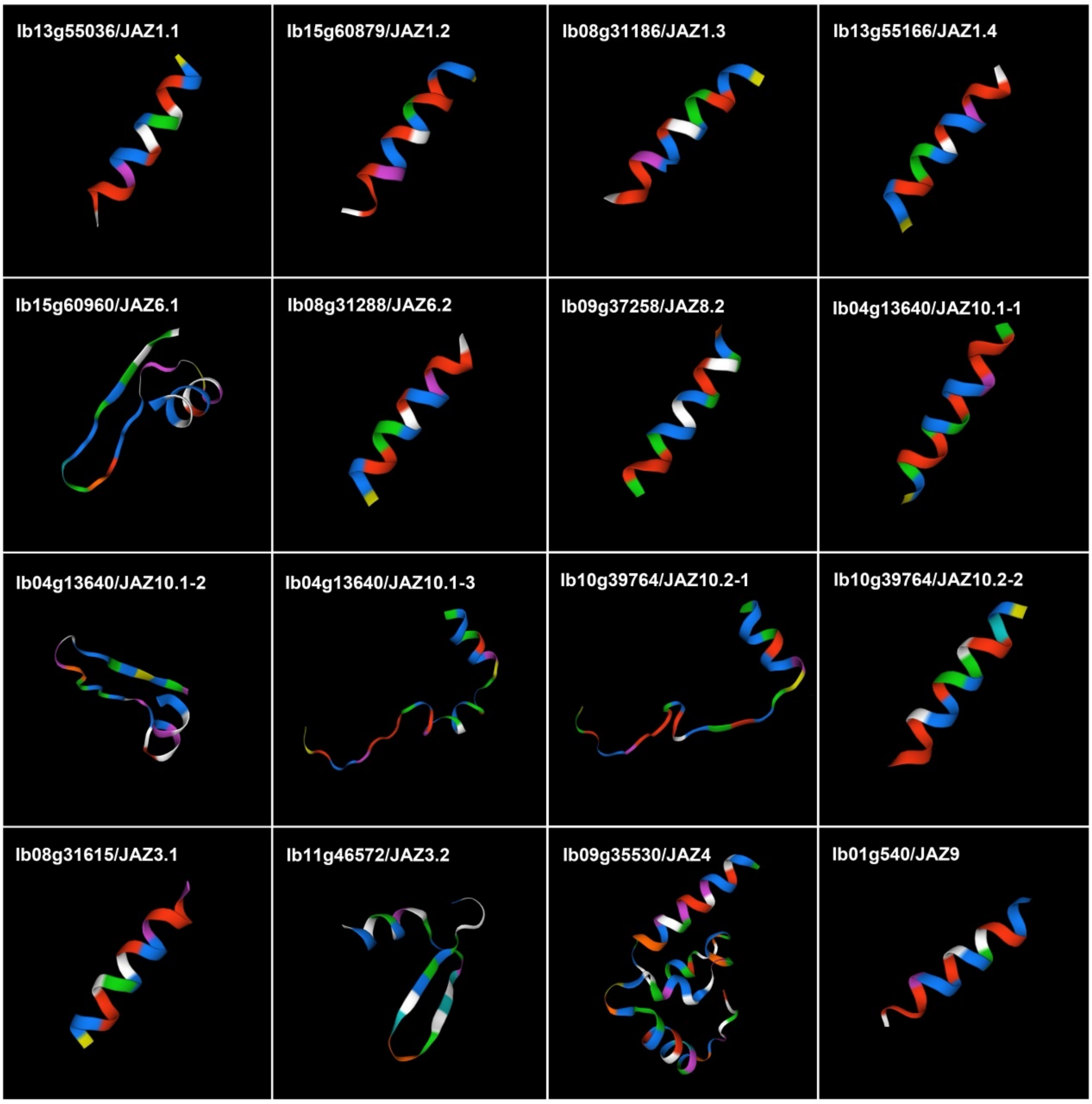

Figure S1. The three-dimensional structural models of IbJAZs in *I. batatas*

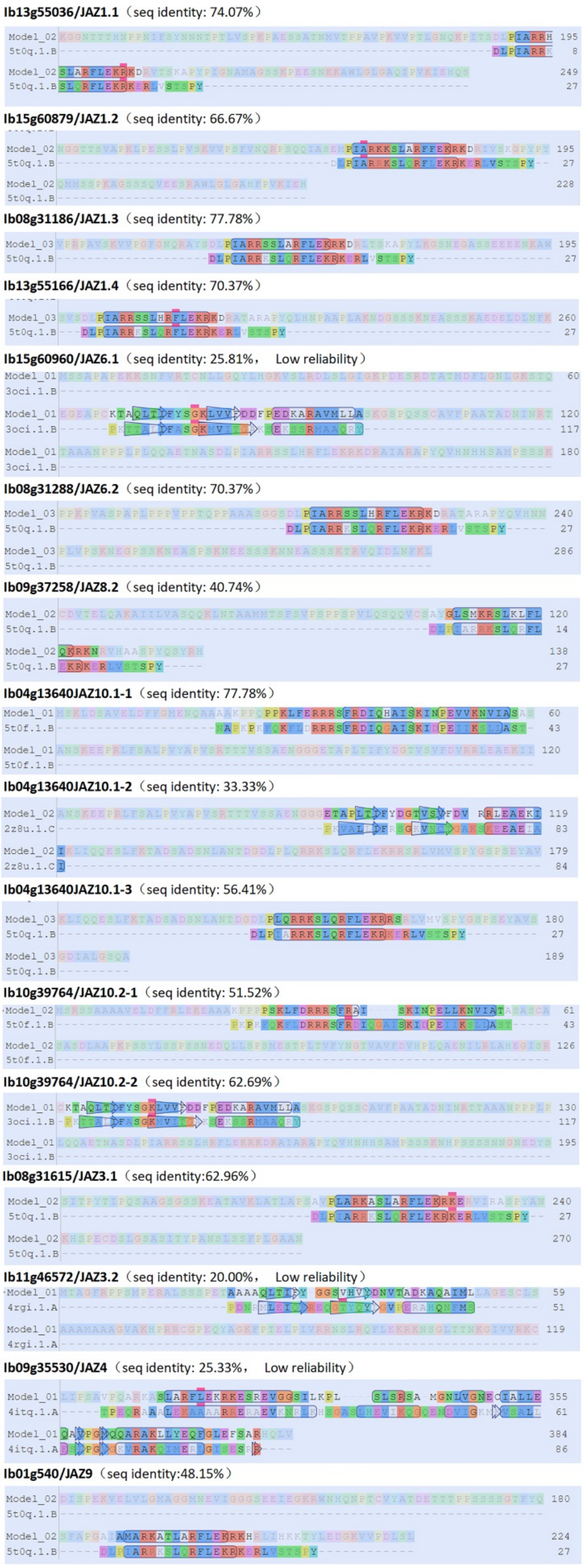

Figure S2. The JAZ proteins sequences used in three-dimensional structural model. The percentage of sequence identity indicted the credibility of the model.

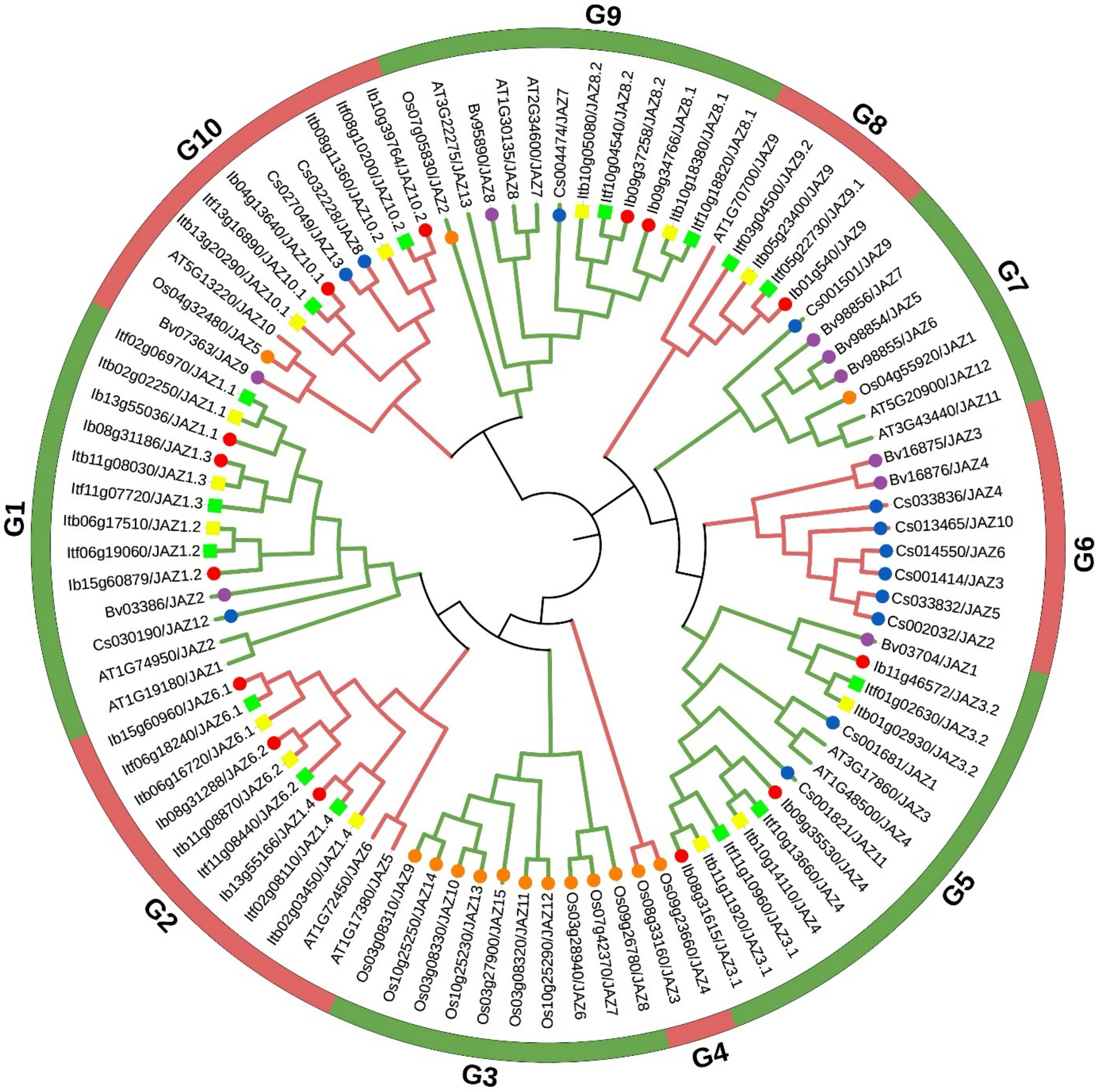

Figure S3. Phylogenetic analysis of the JAZ proteins from seven plant species (i.e. *Arabidopsis*, *B. vulgaris*, *C. sinensis*, rice, *I. batatas*, *I. trifida*, and *I. triloba*). A total of 93 JAZs were divided into ten subgroups (G1 to G10) according to the evolutionary distance. The purple circles, blue circles, orange circles, red circles, green squares, and yellow squares respectively represent the 9 BvJAZs in *B. vulgaris*, 13 CsJAZs in *C. sinensis*, 15 OsJAZs in rice, 14 IbJAZs in *I. batatas*, 15 ItfJAZs in *I. trifida*, and 14 ItbJAZs in *I. triloba*.

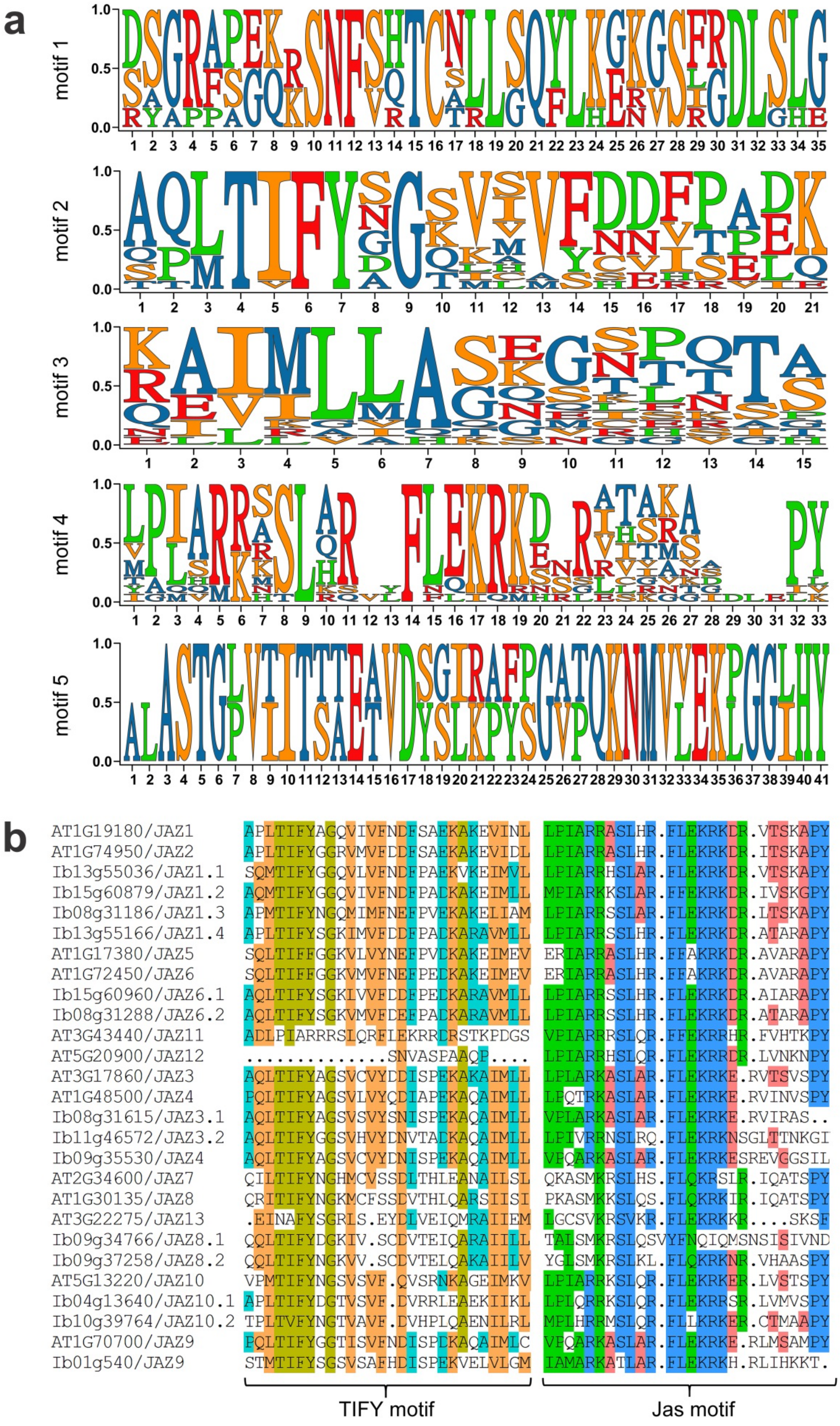

Figure S4. Conserved motifs analysis of IbJAZs in *I. batatas*. (a) Sequence logos of the five conserved motifs. (b) Multiple sequence alignment of TIFY and Jas motifs of IbJAZs.

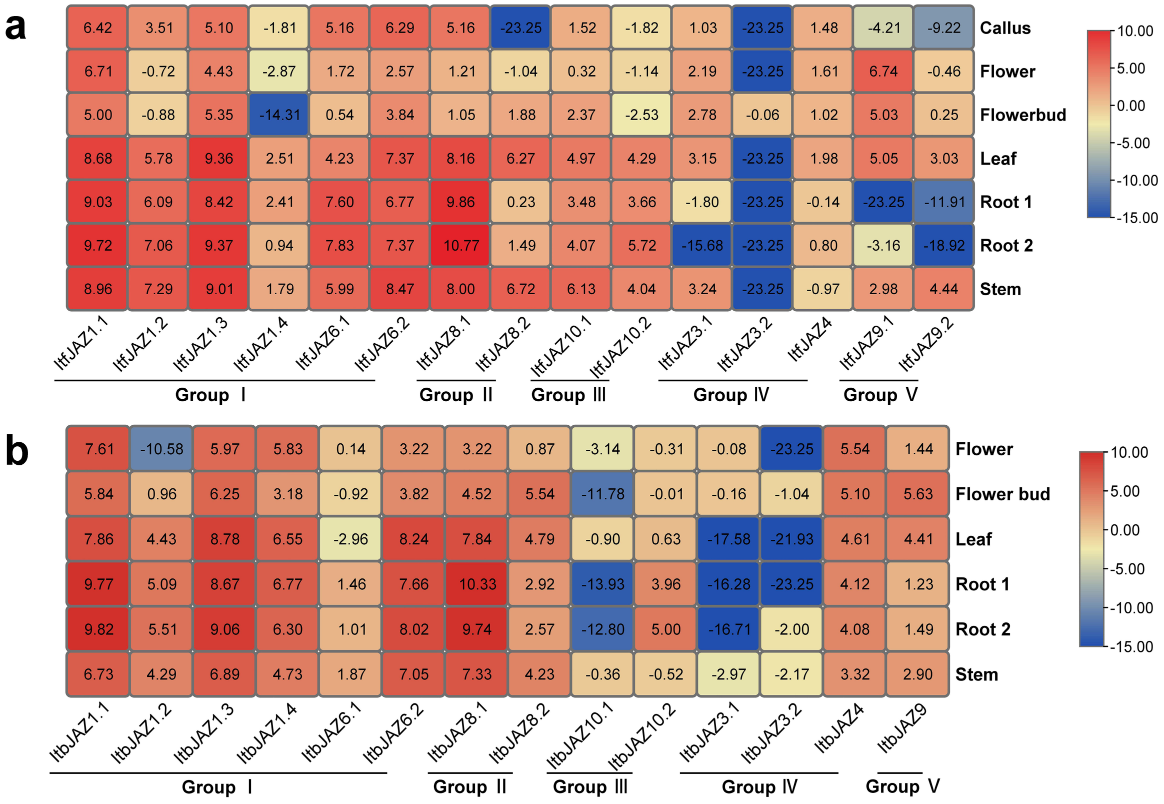

Figure S5. Gene expression patterns of *JAZs* in different tissues of *I. trifida, and I. triloba.* (a) Gene expression patterns of *ItfJAZs* in callus, flower, flower bud, leaf, root 1, root 2, and stem of *I. trifida* as determined by RNA-seq. Log_2 (FPKM) was shown in the boxes. (b) Gene expression patterns of *ItbJAZs* in callus, flower, flower bud, leaf, root 1, root 2, and stem of *I. trifida* as determined by RNA-seq. Log_2 (FPKM) was shown in the boxes.

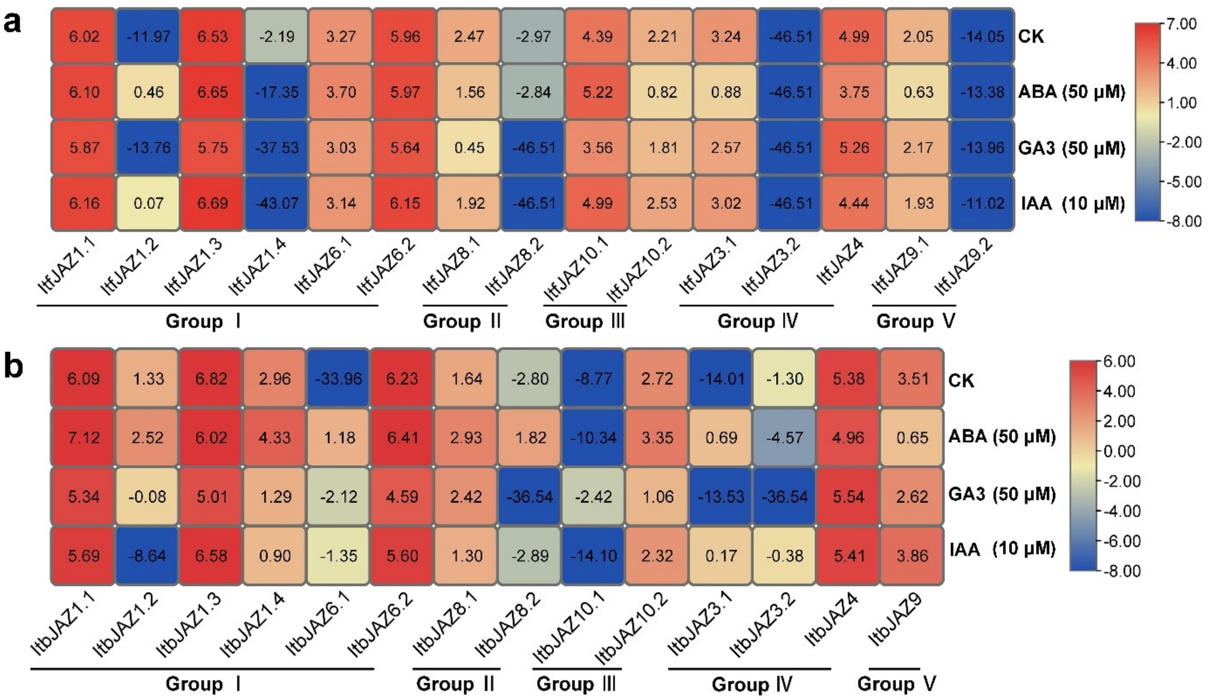

Figure S6. Gene expression patterns of *JAZs* in response to different hormone of *I. trifida* and *I. triloba* as determined by RNA-seq. (a) Expression analysis of *ItfJAZs* under ABA, GA, and IAA treatments in *I. trifida*. (b) Expression analysis of *ItbJAZs* under ABA, GA, and IAA treatments in *I. triloba*. Hormone control: CK. Log2 (FPKM) was shown in the boxes.

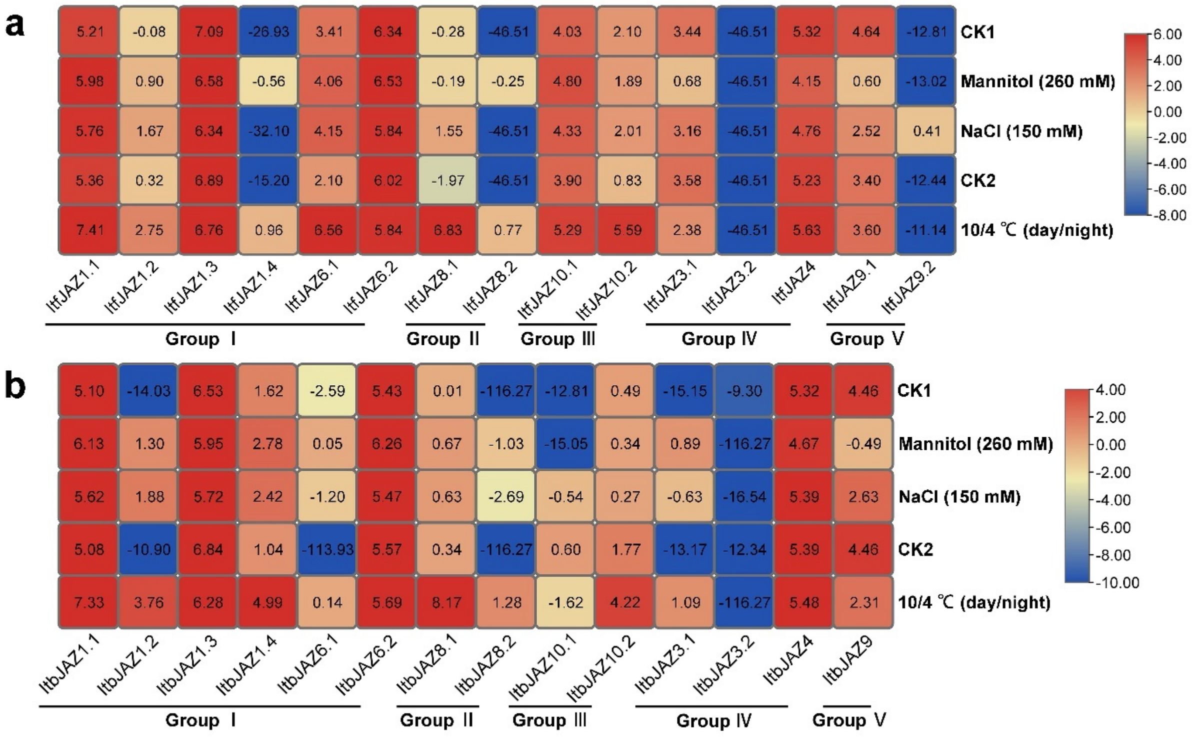

Figure S7. Gene expression patterns of *JAZs* under abiotic stress in *I. trifida* and *I. triloba* as determined by RNA-seq. (a) Expression analysis of *ItfJAZs* under mannitol, NaCl, and 10/4 ℃ (day/night) treatments in *I. trifida*. (b) Expression analysis of *ItbJAZs* under mannitol, NaCl, and 10/4 ℃ (day/night) treatments in *I. triloba*. Mannitol and NaCl control: CK1; cold control: CK2. Log2 (FPKM) was shown in the boxes.
